# Multilineage-differentiating stress-enduring cells: a powerful tool for tissue damage repair

**DOI:** 10.3389/fcell.2024.1380785

**Published:** 2024-05-30

**Authors:** Hanyun Que, Erziya Mai, Yanting Hu, Hong Li, Wenxin Zheng, Yuchen Jiang, Feiruo Han, Xuedong Li, Puyang Gong, Jian Gu

**Affiliations:** College of Pharmacy, Southwest Minzu University, Chengdu, China

**Keywords:** Multilineage-differentiating stress-enduring (Muse), sphingosine-monophosphate (S1P), sphingosine-monophosphate receptor 2 (S1PR2), mesenchymal stem/stromal cells (MSCs), bone marrow (BM), stage-specific embryonic antigen 3 (SSEA-3), fluorescence-activated cell sorting, (FACS)

## Abstract

Multilineage-differentiating stress-enduring (Muse) cells are a type of pluripotent cell with unique characteristics such as non-tumorigenic and pluripotent differentiation ability. After homing, Muse cells spontaneously differentiate into tissue component cells and supplement damaged/lost cells to participate in tissue repair. Importantly, Muse cells can survive in injured tissue for an extended period, stabilizing and promoting tissue repair. In addition, it has been confirmed that injection of exogenous Muse cells exerts anti-inflammatory, anti-apoptosis, anti-fibrosis, immunomodulatory, and paracrine protective effects *in vivo*. The discovery of Muse cells is an important breakthrough in the field of regenerative medicine. The article provides a comprehensive review of the characteristics, sources, and potential mechanisms of Muse cells for tissue repair and regeneration. This review serves as a foundation for the further utilization of Muse cells as a key clinical tool in regenerative medicine.

## 1 Introduction

Currently, there’s a growing necessity to rebuild and substitute damaged tissue, stemming from age-related and other degenerative conditions, tumors, injuries, and birth defects (Sobral-Reyes and Lemos, 2020). Nevertheless, a range of tissue engineering techniques, such as functional biomaterials, drug-eluting systems, and stem cell treatments, have been employed to improve tissue regeneration (Biondi et al., 2008; Shi et al., 2010; Ho et al., 2017). Restoring damaged tissues is vital for survival. Regenerative medicine is an interdisciplinary field that incorporates stem cell-based therapies, tissue generation and repair, and disease modeling (Rosenthal and Badylak, 2016; Spector et al., 2018). Despite many efforts in the past, embryonic stem cells (ESCs), mesenchymal stem/stromal cells (MSCs), induced pluripotent stem cells (iPSCs), and other cells have become key players in regenerative medicine (Trounson and McDonald, 2015; Ratajczak, 2019). Moreover, these cells have been the basis of numerous clinical trials, but problems such as tumorigenicity, immune rejection, remarkable ethical issues, and difficulties in obtaining large numbers of adult stem cells have been exposed in the progress of therapeutic research (Volarevic et al., 2018; Doss and Sachinidis, 2019; Gorecka et al., 2019; Pereira Daoud et al., 2020).

Muse cells, a subset of MSCs known for their self-renewal and diverse differentiation capabilities, can be differentiated into three germ layers with non-tumorigenic and low-telomerase activity (Kuroda et al., 2010; Wakao et al., 2014). When tissues and organs are damaged, enough Muse cells are crucial for maintaining tissue regeneration and functional integrity. It is an ideal seed cell in the fields of tissue engineering, cell transplantation, and gene therapy. It is also the best candidate cell for endogenous repair and may also be the breakthrough for disease treatment (Dezawa, 2016).

Muse cells can selectively accumulate at the site of injury in both circulation and tissue because they express sphingosine-monophosphate (S1P) receptor 2 (S1PR2), which senses the S1P produced by damaged tissue, enabling it to migrate and homing to the site of tissue injury. In contrast to other stem cells, they can be obtained from living organisms, do not require genetic manipulation, and maintain their stem cell potential through naturally occurring mechanisms (Oguma et al., 2022). The most striking advantages are, first of all, the absence of the side effect of the formation of teratomas (Simerman et al., 2014; Li, 2021). Secondly, there is also no induction of host immune rejection during autografting to produce tissue-compatible cells, with little error and minimal immune rejection, and they can also tolerate harsh environments that support their survival in damaged/injured tissues (Kuroda et al., 2010; Aprile et al., 2021). Thus, it plays a key role in tissue healing and regenerative medicine.

This paper reviews its related research (existing mechanism of treatment of disease, clinical research progress), and highlights Muse cells’ potential for clinical application on tissue regeneration and their possible mechanisms of action. Besides, the safety and reliability of Muse cells overcome the defects of most stem cells and are an excellent alternative to ESCs and iPSCs in the field of regenerative medicine. It’s a valuable addition to the toolbox of future clinical treatments for major diseases, offering broad prospects for the treatment of a wide range of clinical diseases.

## 2 The discovery of Muse cells

### 2.1 The source

Dezawa’s group first isolated and discovered a distinct cell subpopulation of MSCs in 2010 in bone marrow (BM) aspirates under prolonged trypsin incubation, which was named Muse cells because of their stress-tolerant properties (Kuroda et al., 2010). Stage-specific embryonic antigen 3 (SSEA-3), a sphingolipid, is a marker for identifying Muse cells and is used to isolate this population from mesenchymal stromal cells (Liang et al., 2010; Sato et al., 2020). Since almost all MSCs are positive for CD 105 (a marker for MSCs), a single application of SSEA-3 will be sufficient to purify Muse cells from MSCs. Muse cells showed expression of CD105 and SSEA3 (a marker for ESCs) double-positive cells, and primitive MSCs were separated into Muse cells (SSEA-3+) and non-Muse cells (SSEA-3−) by either fluorescence-activated cell sorting (FACS) or magnetically-affinitive cell sorting (MACS) (Kuroda et al., 2010; Wakao et al., 2011). Briefly, non-Muse cells (cells other than Muse cells among the MSCs) that do not express SSEA-3 but only express general mesenchymal markers such as CD105, CD29, and CD90 (Kuroda et al., 2010; Kuroda et al., 2013). While Muse cells exhibit not just SSEA-3 expression, but also the pluripotent indicators octamer binding transcription factor 3/4 (Oct3/4), sex-determining region Y box 2 (Sox2), Nanog, and reduced expression 1 (Rex1) (Wakao et al., 2011; Ogura et al., 2014). So, Muse cells can differentiate into endodermal, ectodermal, and mesodermal phenotypes and are capable of self-renewal (Wakao et al., 2011; Wakao et al., 2018a). Therefore, SSEA-3 can be directly applied to separate Muse cells from non-Muse cells.

**FIGURE 1:**
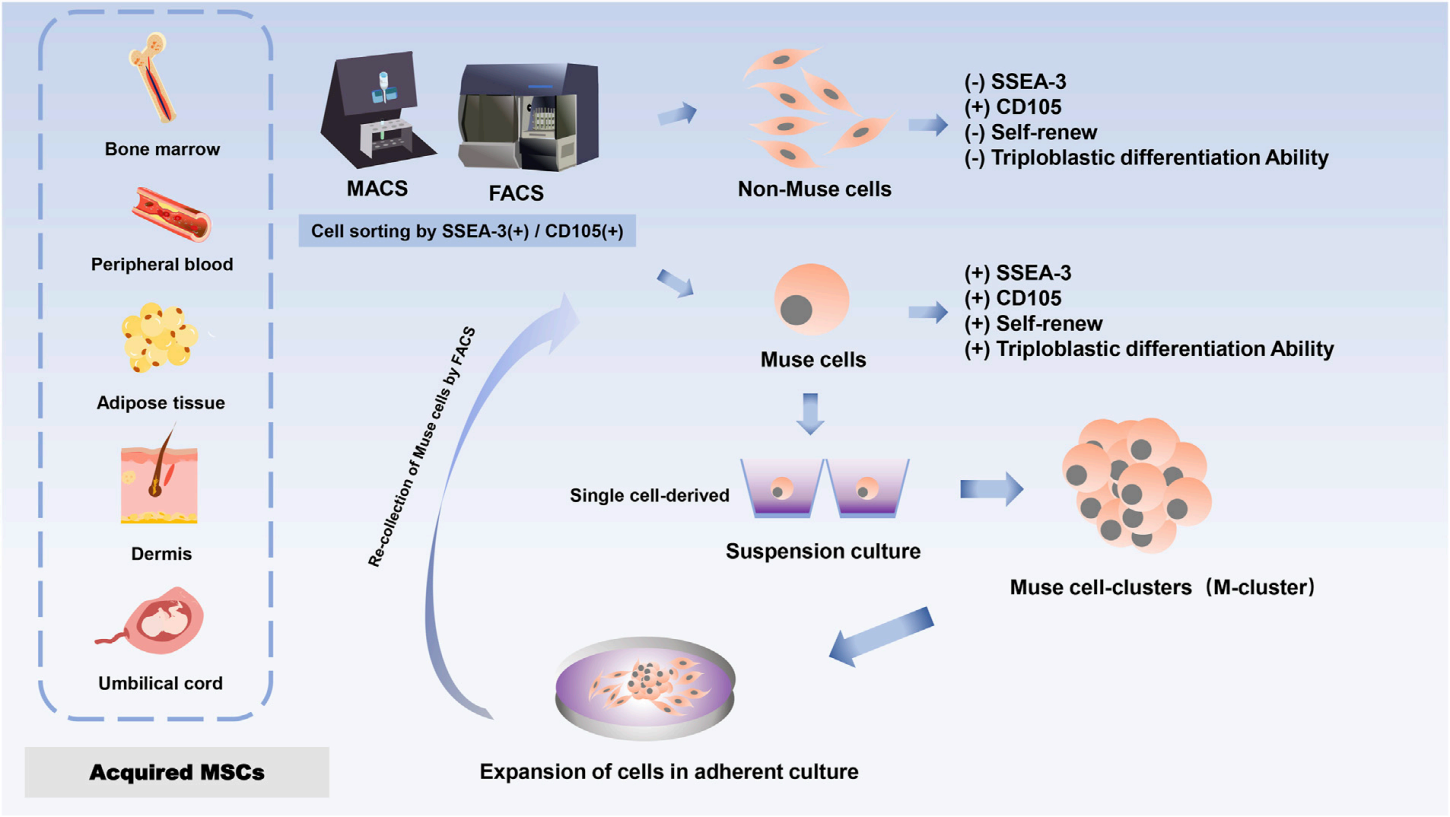
Schematic of Muse cell donor source and expanded cells.

Furthermore, the pluripotency of Muse cells does not need to be induced by the introduction of exogenous genes; they can be isolated from skin and bone marrow obtained from individuals or cell banks (Kuroda et al., 2010). Although each tissue contains a very small number of stem cells and the proportion of Muse cells in bone marine-derived monocytes is small, a large number of Muse cells can be obtained from mesenchymal cell populations through a series of culture steps, such as Muse cell selection, M-cluster formation in suspension culture, and cell amplification in adherent culture (Kuroda et al., 2010; Kuroda et al., 2013) (Figure 1). To date, Muse cells can be successfully isolated from many adult tissues, mainly BM (Kuroda et al., 2010; Kuroda et al., 2013), peripheral blood (PB) (Sato et al., 2020), adipose tissue (AT) (Heneidi et al., 2013; Ogura et al., 2014), dermal fibroblasts (Wakao et al., 2011; Kuroda et al., 2013; Liu et al., 2016), and umbilical cord (UC) (Leng et al., 2019). The most common sources of Muse cells are BM-derived MSCs (BM-MSCs) and UC-derived MSCs (UC-MSCs). The primary source of Muse cells of peripheral blood origin is thought to be the bone marrow, and given that Muse cells in the peripheral blood enter the tissues from the circulatory system, they are first naturally localized to connective tissues and are thought to be continuously mobilized from the bone marrow to the peripheral blood, where they are then supplied to each organ via the bloodstream (Sato et al., 2020; Kikuchi et al., 2022). Once in the organ, they are sparsely distributed throughout the connective tissues of the organ, such as the dermis, liver, spleen, pancreas, trachea, adipose tissue, dental pulp, and synovial tissue (Heneidi et al., 2013; Tsuchiyama et al., 2013; Kinoshita et al., 2015; Dezawa, 2016; Gimeno et al., 2017; Tian et al., 2017; Toyoda et al., 2019; Aprile et al., 2021), so that Muse cells are spread throughout the different mesenchymal tissues. In addition, the functions of Muse cells from different sources are similar. Muse cells that can be isolated and characterized from menstrual blood-derived endometrial stem cells (MenSC) have been reported to provide another source of inspiration (Li et al., 2022).

Thus, the distributional characteristics of Muse cells make them different from other somatic cells, and Muse cells are naturally occurring endogenous cells. Notably, Muse cells found by cell sorting from bone marrow aspirates resulted in a low yield of approximately 1% Muse cells in the total population (8,000 cells/mL initial culture) (Kuroda et al., 2010). In contrast, Muse-AT cell isolation greatly increased this yield capacity through severe cellular stress (Heneidi et al., 2013), opening up the possibility of viable clinical doses of Muse cells in humans.

### 2.2 Characteristics of Muse cell

Importantly, Muse cells are indistinguishable from other predominantly mesenchymal cells in adherent cultures, but when they are transferred to suspension cultures, they form characteristic clusters of cells, are positive for pluripotency markers, and exhibit self-renewal and differentiation (Kuroda et al., 2010; Wakao et al., 2011). It should be noted that Muse cells are a subpopulation of MSCs and can be distinguished from other cells by SSEA-3 (Kuroda et al., 2010). Therefore, Muse cells were positive for both pluripotent and mesenchymal markers, while non-Muse MSCs were positive only for mesenchymal markers (Fisch et al., 2017). In addition, when Muse and non-Muse cells were isolated from MSCs by cell sorting, Muse and non-Muse cells did not differ greatly in cell size (Kuroda et al., 2010). Both sections contain a range of cell sizes, and there is no significant size difference trend. Besides, Muse cells express the pluripotency genes, such as octamer binding transcription factor 3/4 (OCT3/4), sex-determining region Y box 2 (SOX2), Nanog (homeobox protein NANOG), reduced expression 1 (REX1) (Kuroda et al., 2010; Dezawa, 2016), while cells other than Muse cells containing MSCs did not express pluripotent genes and did not cross the oligonucleotide boundaries between mesoderm, ectoderm, and endoderm (Heneidi et al., 2013; Ogura et al., 2014; Simerman et al., 2014; Simerman et al., 2014).

Oct3/4, SOX2, and Nanog are embryonic stem cell markers, which are also core transcription factors maintaining cell pluripotency. Single Muse cells could form ESCs-like clusters in suspension, showing triploblastic differentiation potential and self-renewability, while single non-Muse cells could not survive in suspension and self-renewal is not feasible in (Kuroda et al., 2010). Because Muse cells can differentiate into mesodermal cells [skeletal muscle (Ee et al., 2017), cardiomyocytes (Amin et al., 2018), glomerular cells (Uchida N. et al., 2017)], endodermal cells [hepatocytes (Iseki et al., 2017), bile duct cells (Fukase et al., 2022)], and ectodermal cells [melanocytes (Tian et al., 2017), nerve cells (Yamashita et al., 2021), keratinocytes (Yamauchi et al., 2017)], while the differentiation ability of non-Muse MSCs was limited to adipocytes, bone cells, and chondrocytes, and their differentiation ratio was lower than that of Muse cells (Dezawa, 2016).

### 2.3 Adhesion-suspension switch may control Muse cell pluripotency

Muse cells were obtained from cultured MSC by FACS. Such cells proliferate asymmetrically in the adherent culture state, giving rise to a new Muse cell in addition to a non-Muse cell. The flat and elongated non-Muse cells surround the Muse cells to form a sheath. The sheathed Muse cells then proliferate by symmetric division to produce mature clusters of 50–150 μm in size within 2 weeks. When mature clusters were cultured in an adherent state, internal Muse cells proliferated by asymmetric division after moving out of the cluster (Kushida et al., 2018a). As a result, the number of Muse cells gradually decreases, eventually accounting for only a certain percentage of the total number of cells, which is consistent with the proportion of Muse cells in MSCs and fibroblasts (Kuroda et al., 2010).

It is worth mentioning that the pluripotency of Muse cells is regulated by an “adherence-suspension switch,” which is different from MSCs because Muse cells can survive and proliferate in both adherent and suspension states (Kushida et al., 2018b). Adhesion-suspension switch may control Muse cell pluripotency. The “adherence-suspension switch” is involved in the control of Muse cell pluripotency. Some researchers found that Nanog, SOX2, Oct3/4, transcription factors that maintain stem cell pluripotency, are distributed in the cytoplasm when they are attached to the wall, while in suspension culture they are present in the nucleus, thus explaining why the expression of these genes is 50 to hundreds of times higher in suspension than in walled culture (Kuroda et al., 2010; Ogura et al., 2014; Iseki et al., 2017). Moreover, the expression levels of these pluripotent genes are reversible between adherent and suspension states, which alter the epigenetics of Muse cells (Iseki et al., 2017).

Therefore, Muse cells in the organ’s connective tissue, including the Muse cells in the bone marrow, are considered to have lower pluripotency. The function of Muse cells in the resting state is not stimulated, at this point, the cell is in an adherent state, but the stimulated Muse cells will exert their function. In the organism, once the Muse cells are mobilized into the peripheral blood and kept in suspension, their pluripotent factors are activated and highly activated, and the suspension environment enhances the pluripotency of the Muse cells. Pb-Muse cells are considered to have high pluripotency due to the significantly increased pluripotency of Muse cells in suspension (Sato et al., 2020). Indeed, Muse cells in different tissue-derived, the core characteristics, the expression of pluripotent genes, and the ability of triploid differentiation and self-renewal at the individual cell level are consistent. Interestingly, Muse cells show their differentiation direction according to their origin, it may be related to the mobilization of Muse cells into peripheral blood circulation after tissue injury. Amin et al. (2018) treated Muse cells with DNA methylation inhibitors to increase pluripotent gene expression levels in suspension. This provides a bright idea for improving the pluripotency of Muse cells in the later stage. Nevertheless, the relationship between the suspension state of Muse cells and the methylation state of pluripotent genes is unknown. The molecular mechanisms by which adherence-suspension switches control the localization and expression of pluripotent genes still require further investigation.

A study comparing the pluripotent gene expression of human ESC/Induced pluripotent stem cells (iPSCs) and human Muse and non-Muse cells found that the pluripotent gene expression patterns of Muse cells and ESC/iPSCs were very similar. Importantly, non-Muse cells did not express pluripotent genes (Wakao et al., 2011). This is in stark contrast to the Muse cell. Moreover, although the gene expression patterns of cell cycle-related factors (i.e., Tumorigenesis factors) were different between ESC/iPSCs and Muse cells, these tumorigenic factors were generally highly expressed in ESC/iPSCs, while they were very low expressed in Muse cells, and their levels and patterns were similar to those of non-Muse cells, which highlighted Muse cells have a low risk of tumorigenesis (Wakao et al., 2011).

It has been reported that Muse cells can detect DNA damage quickly and activate the DNA damage repair system better than MSCs and non-Muse cells, these two types of cells were treated with H_2_O_2_ and ultraviolet light to induce DNA damage. Then, cells were collected for DNA damage response (DDR) analysis at 1, 6, and 48 h after stress treatment. Finally confirmed that Muse cells showed better resistance to physical and chemical genotoxic stress than non-Muse cells. Although the efficiency of the single-strand repair system was equal in both populations, the double-strand repair system (non-homologous terminating) of Muse cells was more powerful than that of non-Muse cells. Hence, the high ability of Muse cells to cope with genotoxic stress was related to its rapid and efficient sensing of DNA damage and activation of the DNA repair system. In previously reported models of fulminant hepatitis, skeletal muscle degeneration, stroke, and skin regeneration, Muse cells actively migrated to and integrated into the damaged site with higher efficiency than non-Muse cells (Tsuchiyama et al., 2013; Katagiri et al., 2016; Uchida et al., 2016; Ee et al., 2017). In addition, Muse cells were able to return to damaged tissues and survive after integration, while non-Muse cells were not. Thus, Muse cells can work as repairing cells for a wide range of tissues and organs.

Indeed, when Muse cells are fully utilized, the low homing rate of intravenous MSCs will be greatly improved because Muse cells homed to the injury site at a higher rate than MSCs due to their ability to sense injury signals (Dezawa, 2016; Katagiri et al., 2016). In light of the above, the differences between Muse and non-Muse cells are shown in Table 1.

**TABLE 1:** The similarities and differences between Muse cells and non-Muse cells.

Distinction	Muse cells	Non-Muse cells
Tumorigenicity	No	No
Telomerase activity	Low	Low
SSEA3 expression	Yes	No
CD105 expression	Yes	Yes
Nanog, Oct3/4 and SOX_2_ expression	Yes	No
Stress tolerance	High	Low
Self-renew	Yes	No
Pluripotent genes expression	Yes	No
Triploblastic differentiation ability	Yes	No
Survivability in adhesion/suspension	Both	Adhesion
Migration toward damaged tissue by intravenously injection	Yes	No
Spontaneous differentiation compatible *in vivo*	Yes	No
Cell differentiation before transplantation	No	No

## 3 Muse cell is an ideal regeneration tool

### 3.1 No tumorigenic risk has been identified

Pluripotent cells are highly expected to contribute to regenerative medicine because of their ability to differentiate into any type of cell in the body (Ratajczak, 2019), which means they could be applied to a wide variety of diseases. Muse cells are involved in the multi-lineage differentiation of MSCs. Meanwhile, a single Muse cell can generate cells representing each of the three germ layers and has the ability to self-renew at the single-cell level (Kuroda et al., 2010; Rs et al., 2010). As Muse cells are different from normal ESCs and iPSCs transplanted, Muse cells are naturally occurring in organisms and are autologous, they do not require cytokine pre-treatment before administration or introduction of genes into the cells for differentiation purposes and there is little to no erroneous replacement of damaged/apoptotic cells after homing into damaged tissues (Kuroda et al., 2010; Simerman et al., 2014; Kushida et al., 2018b). The process progresses rapidly compared to *in vitro* cytokine-induced differentiation.

Most importantly, Muse are non-tumorigenic, consistent with the fact that they are present in the body. Pluripotency is both a curse and a blessing for stem cells, as the ability of the three germ layers to differentiate and self-renew is often uncontrolled, often resulting in the formation of teratomas. Telomerase activity remains high in stem cells, immortalized cancer cells, and ESCs/iPSCs, possibly to support their proliferation and self-renewal. Telomerase activity is also used as a marker of tumorigenic activity (Huang et al., 2011; Ogura et al., 2014; Liu et al., 2016). The discovery of iPSCs is earlier than Muse cells, and there are many clinical studies. Although unlimited proliferation is the advantage of iPSCs, it has also become a defect that teratomas are easily formed *in vivo* after transplantation (Yamanaka, 2020).

On the other hand, the Lin28 gene plays a key role in maintaining the pluripotency of these two types of cells (ESCs and iPSCs) and in generating tumors, and Let-7 is a microRNA that regulates embryonic development, cell differentiation, and tumor inhibition (Thornton and Gregory, 2012), this phenomenon is thought to prevent tumor formation and promote tissue regeneration (Simerman et al., 2016). Unlike ESCs/iPSCs, the pluripotency of Muse cells does not depend at all on the tumor-prone protein Lin28, but rather the process through which the tumor-inhibiting microRNA let7 sustains pluripotency has been uncovered. Research on Muse cells revealed that let-7 suppresses the PI3K-AKT route, resulting in the continuous activation of the crucial pluripotency controller KLF4 and its subsequent genes, POU5F1, SOX2, and NANOG. Let-7 further hindered cell growth and glycolysis through the suppression of the PI3K-AKT pathway, indicating its role in promoting non-cancerous growth. Additionally, let-7 does not regulate the MEK/ERK pathway, which could play a crucial part in sustaining self-renewal and curbing aging (Wakao et al., 2011; Li et al., 2024). It’s possible that Muse cells possess a distinct protective strategy to inhibit the excessive production of let-7, a concept yet to be clarified. Interestingly, while the proliferation of ESCs and iPSCs is dependent on leukemia inhibitory factor (LIF) and bone morphogenetic protein 4 (BMP4), in contrast, Muse cells are dependent on a family of fibroblast cytokines to maintain their self-renewal and proliferative capacity, which may also explain why it is not tumorigenic (Wakao et al., 2018a). From this, it is clear that Muse cells have evolved multiple fail-safe mechanisms to avoid growing themselves out of control. Previous studies have reported that Muse cells were transplanted into the testicles of immunodeficient mice with Muse iPS cells (treated Muse cells confer iPS properties) that did not form teratomas for up to 6 months, while the formation of teratomas after 12 weeks of implantation of Muse-iPS cells. The result indicated that non-tumorigenic Muse cells were induced by iPS cells to gain tumorigenic proliferative activity (Wakao et al., 2011). Nowadays, there are no reports of side effects such as tumorigenicity after Muse cell transplantation *in vivo*. Based on animal models of existing diseases including acute myocardial infarction (AMI) (Minatoguchi et al., 2018; Tanaka et al., 2018), stroke (Hori et al., 2016; Uchida H. et al., 2017), kidney fibrosis (Iseki et al., 2017), and other diseases (Hosoyama and Saiki, 2018; Perone et al., 2018; Fujita et al., 2021; Fukase et al., 2022), Muse cells not only stimulate tissue regeneration and recovery but also show that they do not form tumors *in vivo* after transplantation.

It is important to note that Muse cells can be administered intravenously, eliminating the need for surgery and reducing associated risks. Furthermore, they are a safe choice for clinical regeneration.

### 3.2 Matching or immunosuppressive therapy

It is well known that an issue that should be considered in allogeneic therapy is the immune response of the recipient after transplantation. This response has been recognized in organ and hematopoietic transplantation, so the use of immunosuppression is needed to protect allogeneic grafts from rejection (Chinen and Buckley, 2010). It is also important to consider that *in vivo* infusion or transplantation of allogeneic cells without appropriate HLA or major histocompatibility complex (MHC) matching or the use of immunosuppression, the problem of cell rejection by the host’s immune system can quickly arise (Chinen and Buckley, 2010; Ankrum et al., 2014; Kot et al., 2019). In conclusion, immune rejection is the greatest challenge to allogeneic cell therapy (Zhao et al., 2019).

Surprisingly, allogeneic Muse cell transplants are free of immune rejection, eliminating the need to acquire immune tolerance through autologous or allogeneic transplantation (Young, 2018). Donor-derived allogeneic Muse cells have the very beneficial feature of being administered directly to the patient without the need for HLA-matching and immunosuppressive treatment. Donor-derived Muse cells can migrate directly into patients without HLA-matching and immunosuppressive treatment (Dezawa, 2018a; Tanaka et al., 2018; Young, 2018). In a study of Muse cell entry into infarcted rabbit hearts, Muse cells expressed HLA-G higher than MSCs and were found that intravenously infused Muse cells could survive as differentiated cells in host tissues for more than 6 months, even without immunosuppressive treatment. This anti-immune property makes Muse cells useful not only in tissue repair but also in suppressing autoimmunity (Yamada et al., 2018). HLA-G is a mechanism evolved by placental mammals to prevent immune rejection of the placenta and the fetus it nurtures (Lila et al., 2002; Tipnis et al., 2010; Kot et al., 2019). Muse cells’ immunological advantage is partially attributed to the presence of HLA-G, found in placental extravillous trophoblast cells, crucial for immune tolerance in pregnancy. HLA-G has a strong immunosuppressive effect, effectively inhibiting the proliferation and maturation of maternal macrophages, T cells, B cells, NK-cells, dendritic cells, and neutrophils (Ankrum et al., 2014; Dezawa, 2016; Kot et al., 2019); therefore, the expression of HLA-G may have a protective effect on Muse cells. Whereas Indoleamine 2,3- dioxygenase (IDO) inhibits the kynurenine pathway by promoting the degradation of tryptophan in T cells, thereby inhibiting T cell proliferation and inducing apoptosis (Tipnis et al., 2010). IDO is also involved in the maturation of regulatory T cells (Treg), which are necessary for the acquisition of immune tolerance, and it has been shown that Muse cells produce IDO at levels similar to MSC (Tipnis et al., 2010; Uchida H. et al., 2017).

**FIGURE 2:**
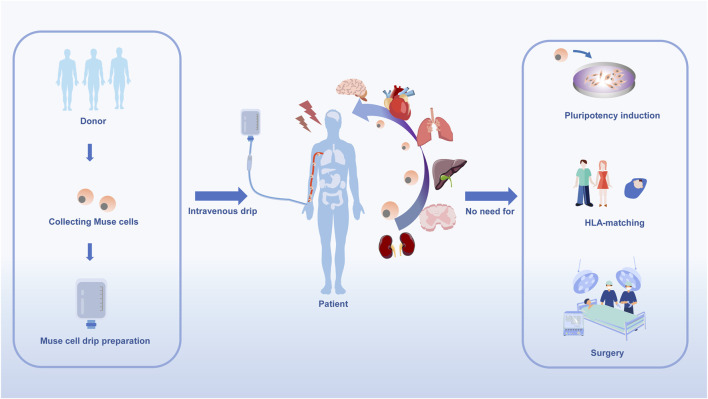
Muse cells collected from tissue sources, expanded and injected intravenously into patients. Since Muse cells are pluripotent-like cells, they may be able to target a variety of diseases.

Muse cells display distinct traits absent in MSCs. Muse cells are distinctively characterized by their ability to specifically target damaged tissues post-intravenous injection, unlike MSCs which remain confined in the lungs, their capacity to differentiate from various damaged/apoptotic cells via phagocytosis, and their enduring immunotolerance for the use of donor cells (Minatoguchi et al., 2024). Briefly, the powerful anti-immune mechanism of Muse cells is due to the high expression of HLA-G and the immunomodulator IDO to suppress cellular and humoral immunity (Figure 2). Muse cells may be used as immunomodulators to treat immune-related diseases.

### 3.3 Superior survivability *in vivo*

In the field of regenerative medicine, the use of stem cells is often limited by low survival rates, which often do not exceed 3% when exposed to high-stress transplantation environments (Tower, 2012). Stem cells may be subjected to multiple rounds of internal and external stresses and therefore must have robust and efficient DNA damage checkpoints and DNA repair mechanisms to promote full cellular recovery rather than triggering senescence and/or apoptosis in the event of a genotoxic event (Hodgetts et al., 2000; Tower, 2012; Alessio et al., 2018). Muse cells can be isolated by severe cellular stress conditions, including prolonged exposure to the protein hydrolase collagenase, serum deprivation, hypothermia, and hypoxia (Kuroda et al., 2010; Kuroda et al., 2013; Wakao et al., 2011; Ogura et al., 2014; Dezawa, 2018b). At the same time, Muse cells display highly conserved cellular mechanisms essential for cell survival and proliferation in response to extreme cellular stress (Simerman et al., 2016).

Serpins are superfamily proteins that inhibit trypsin, thrombin, and neutrophil elastase with protease inhibitory activity (Aymonnier et al., 2021; Kellici et al., 2021). Comparing the secretion sets of Muse cells, BM-MSCs, and adipose-derived MSCs (AD-MSCs), Serpins were only expressed in Muse cells, but not in BM-MSCs and AD-MSCs (Wakao et al., 2018b; Kellici et al., 2021). This may explain the high tolerance of Muse cells to prolonged pancreatic enzyme incubation (Kuroda et al., 2010). Moreover, most of the 14-3-3 isomers were involved in anti-apoptotic activity in the Muse cell secretion group (Alessio et al., 2017). The 14-3-3-3 protein is a highly conserved family of 30 kDa molecules that form stable homo-and heterodimers (Clapp et al., 2012). Accumulating evidence suggests that 14-3-3 protein plays a particularly important role in the activation, maintenance, and release of G1/S and G2/M cell cycle checkpoint activation. Besides, the 14-3-3 protein also plays a crucial role in regulating the response to DNA damage after intracellular and extracellular injury (Gardino and Yaffe, 2011; Clapp et al., 2012; Munier et al., 2021; Obsilova and Obsil, 2022). If cell damage occurs, the 14-3-3 protein prevents mitosis from entering the cell by regulating cyclin-related protein kinases and phosphatases (Clapp et al., 2012). Therefore, Muse cells are stress-resistant, and their active secretion of pro-survival factors such as 14-3-3 proteins and serpins, may be the reason why they can survive in the hostile microenvironment of damaged tissues. These factors play a key role in regulating the cell’s response to DNA damage after internal or external damage. At the same time, it also reduces cell stress and subsequent damaged cell apoptosis.

Available studies show that human Muse cells survive as physiologically active cardiomyocytes in post-infarct cardiac tissue for 2 weeks after administration in rabbits (Yamada et al., 2018). Similarly, human Muse cells were found to survive for 4 weeks in chemically induced Hunner-type interstitial cystitis-like rats (Furuta et al., 2022). The harsh microenvironment of lung ischemia-reperfusion (IR) injury contains both pro-apoptotic and pro-inflammatory cytokines and reactive oxygen species (Yabuki and Watanabe, 2018; Chen et al., 2022). In a study of acute lung IR injury in a rat model, the results showed that human Muse cells were more effective at homing into damaged lung tissue, inhibiting apoptosis and promoting proliferation of host alveolar cells than MSCs (Yabuki and Wakao, 2018). In certain diseases, such as stroke, myocardial infarction or renal failure, there is a high level of apoptosis and degeneration of tissue cells. This leads to a very stressful environment in the body and stem cell therapies may fail as the stem cells may be damaged before they can play a regenerative role. However, Muse cells show a strong ability to sense and survive DNA damage in these diseases and play a reparative role (Uchida N. et al., 2017; Amin et al., 2018; Minatoguchi et al., 2018; Uchida et al., 2018; Li et al., 2022).

In summary, Muse cells have a very active anti-stress and anti-cellular transformation protection mechanism, which is undoubtedly a very important property that contributes to the maintenance of their function and the promotion of tissue and organ homeostasis.

### 3.4 Tissue-protection effects

When tissues are damaged, the damage persists due to the inflammatory response in the microenvironment surrounding the injury that exacerbates apoptosis at the site of injury. Muse cells are able to spontaneously differentiate into cells compatible with their tissues (Katagiri et al., 2016; Kushida et al., 2018b), leading to robust tissue repair by replenishing functional cells. Indeed, Muse cells replace damaged/dead cells by differentiating into tissue-forming cells *in vivo*, which are immunomodulated and release anti-inflammatory, anti-apoptotic, and anti-fibrotic-related factors for tissue protection.

Due to the Muse cell’s pluripotent differentiation capacity, Muse cell-based therapy has been explored in a broad range of diseases. In a study of cell fate and function of human skin fibroblast-derived Muse cells were evaluated in a rat stroke model (Uchida et al., 2016), they differentiate with a high ratio into neuronal cells after integration with host brain microenvironment, possibly reconstructing the neuronal circuit to mitigate stroke symptoms. Muse cells not only home to damaged tissues, they also directly participate in the formation of new blood vessels by spontaneously differentiating into vascular cells, a process that involves the production of neovascularization activators VEGF and HGF, as shown by typical animal models of acute myocardial infarction (AMI) (Yamada et al., 2018), liver damage (Hessheimer et al., 2021a; Shono et al., 2021) and aortic aneurism (Hosoyama et al., 2018). Thus, Muse cells have roles in both vascular protection and neovascularization. The above studies have shown that it can spontaneously differentiate into three germ layer lineages adapted to the tissue microenvironment, thereby protecting damaged tissue. The potent anti-inflammatory effects of Muse cells are augmented by the ability to survive for a long period as integrated cells in host tissues, whether autologous or allogeneic (Hodgetts et al., 2000; Dezawa, 2016). Macrophages also significantly reduced tumor necrosis factor-α (TNF-α) production when co-cultured with Muse cells *in vitro*. Muse cells had a significant protective effect on the proliferative maintenance of TNF-α-injured intestinal epithelial crypt cells and on the intestinal barrier structure by decreasing the secretion of IL-6 and IFN-γ and increasing the release of TGF-β and IL-10 in the inflammatory microenvironment (Sun et al., 2020). In rat models of interstitial cystitis (Furuta et al., 2022) and severe pancreatitis (Fouad et al., 2018; Fukase et al., 2022), the administration of Muse cells significantly inhibited the infiltration of inflammatory cells such as macrophages and neutrophils and effectively reduced oedema at the site of injury, thereby protecting the tissues against further damage.

Muse cells survive in host tissues and remain integrated for an extended period of time, and their anti-fibrotic, anti-inflammatory, anti-apoptotic and paracrine effects are correspondingly long-lasting and effective. Thus, the pleiotropic nature of Muse cells allows them to exert potent tissue-protective effects, and their unique ability to provide viable therapeutic approaches for many diseases.

## 4 Muse cell repairs the location of the damage

### 4.1 The S1P-S1PR2 system is the main axis that controls the selective homing of circulating Muse cells

**FIGURE 3:**
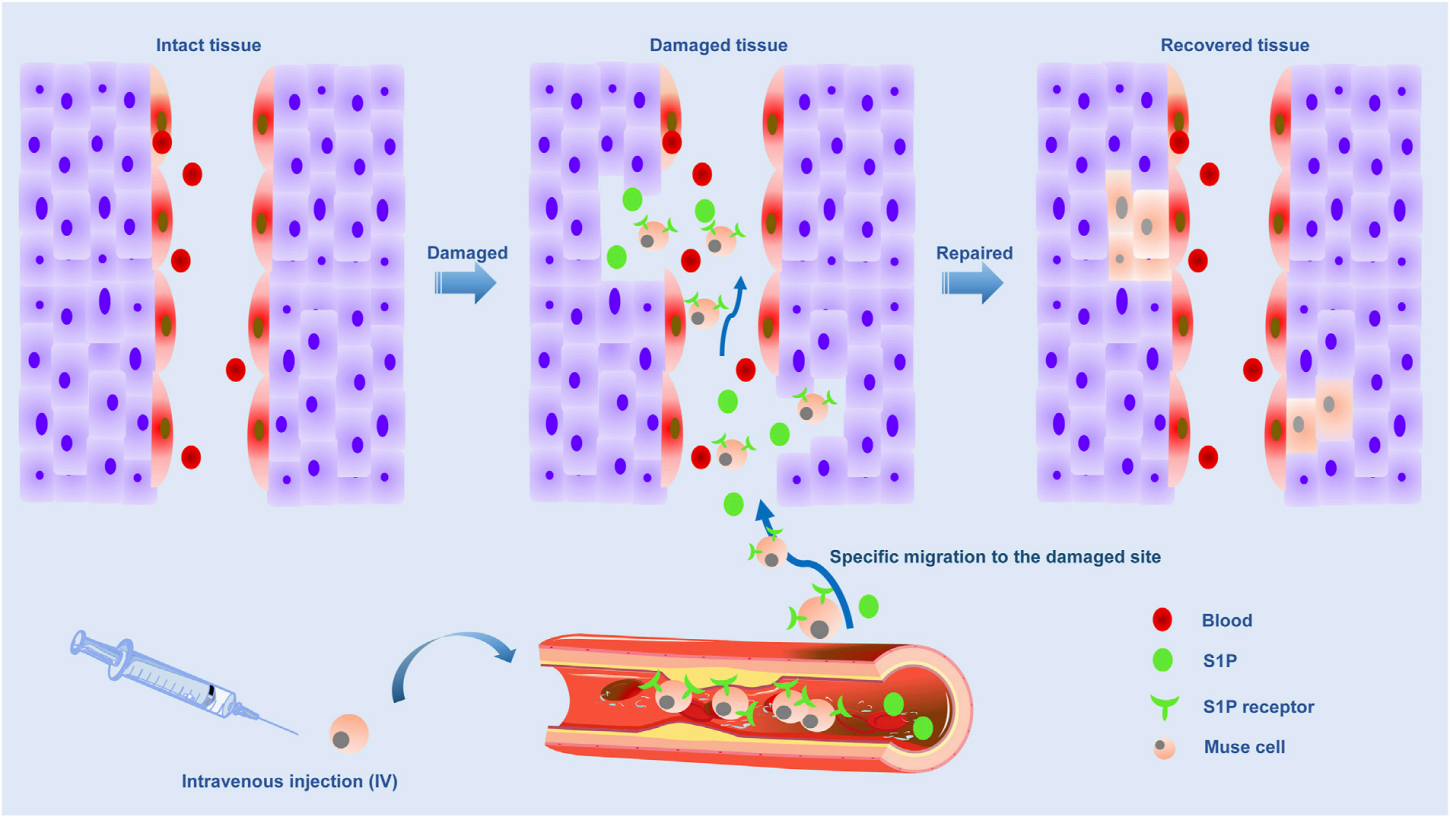
Schematic representation of the repair role of Muse cells through the S1P-S1PR2 axis to the injury site.

The ability to strongly perceive damage signals released by injured/damaged tissues is a unique and prominent feature of Muse cells due to the selective accumulation of damage sites mediated by S1P-S1PR2-axis (Yamada et al., 2018). S1P is an alarm signal of acute inflammation/injury and is actively produced by damaged cells by phosphorylating S1P, a cell membrane component (Weigert et al., 2019). In a word, when cells are damaged, S1P is produced. There are five subtypes of S1P receptors, including S1PR1, S1PR2, S1PR3, S1PR4, and S1PR5 (Obinata and Hla, 2019). Previous studies have found that S1P is involved in the proliferation, movement, morphology and differentiation of tumor cells, neurons, vascular smooth muscle cells and vascular endothelial cells, which is associated with S1P receptors (Lee et al., 1998; Okamoto et al., 1998; Du et al., 2010; Obinata and Hla, 2019; Weigert et al., 2019). Muse cells express S1PR2, allowing them to keenly sense S1P signals produced by damaged tissue and selectively return to the site of injury where they have accumulated. Therefore, Muse cells were guided to migrate to the site of injury by the S1P-S1PR2 system. Once tissue injury occurs, Muse cells will be mobilized from bone marrow to peripheral blood, and endogenous and exogenous Muse cells (in the case of transplantation) return to the injured site via the S1P-S1PR2 system (Yamada et al., 2018) (Figure 3).

Based on clinical data from patients with acute myocardial infarction and stroke, the level of S1P in serum increased before the number of endogenous PB-Muse cells increased. Shortly after injury, S1P was released into the peripheral blood, and this increase in the acute phase was significantly associated with functional recovery at 6 months, supporting the repair function of Muse cells *in vivo* (Hori et al., 2016; Tanaka et al., 2018; Yamada et al., 2018). In acute myocardial infarction (AMI), S1P is produced from the infarct area as an alarm signal and transmitted to the bone marrow, where S1P is mobilized to the peripheral blood to increase the number of circulating Muse cells. In addition, circulating Muse cells migrated axially to infarct area via S1P-S1PR2, and replaced damaged cells through spontaneous differentiation into tissue suitable cells to repair heart tissue. When the number of endogenous Muse cells was insufficient, intravenous injection of exogenous Muse cells enhanced the repair activity, leading to successful tissue repair (Tanaka et al., 2018). Muse cells were inhibited by S1PR2 antagonists to migrate to myocardial infarction sections in vitro and myocardial infarction tissues *in vivo*. Compared with unsilenced Muse cells, Muse cells migrated to S1PR2-specific agonist SID 46371153 and AMI heart tissue, suggesting Muse cells migrated through S1P-S1PR2 axis, which was a “targeting” effect (Yamada et al., 2018). These findings support the central role of the S1P-S1PR2 axis in Muse cell-specific homing. At the same time, S1PR2 antagonist also showed that the therapeutic effect of Muse cells was weakened.

It was found that serum S1P level was positively correlated with the number of Muse cells in patients with acute myocardial infarction, is suggesting that patients with more Muse cells in peripheral blood but less Muse cells in acute stage had improved left ventricular function and remodeled in chronic stage (Tanaka et al., 2018). Therefore, the number of Muse cells can predict the prognosis of AMI patients.

### 4.2 The escape of Muse cells from pulmonary capillaries might be key to their positioning at the site of tissue injury

On the base of a great deal of reference literatures, it has been found that when MSCs are injected intravenously into the recipient body, most of them are trapped in the pulmonary capillaries (Barbash et al., 2003; Li et al., 2008; Brooks et al., 2018; Mercer-Smith et al., 2021). In contrast, the majority of Muse cells were able to escape from the coating of lung capillaries because of Muse cells were able to selectively cluster at the site of injury by keenly sensing S1P alarm signals, a key mediator of inflammation, rather than being trapped in lung capillaries and homing in damaged tissue during intravenous injection (Yamada et al., 2018).

Consistently, the ability to spontaneously differentiate into cells compatible with homing tissues *in vivo* after integration, even crossing oligonucleotide boundaries from mesoderm to endoderm or between ectoderm cells, is not recognized by other types of stem cells, including ESC/iPSCs and somatic stem cells such as neural and hematopoietic stem cells (Mercer-Smith et al., 2021). In addition to intravenous injection, Muse cells can also be injected locally to the site of injury. In the study of Yamauchi et al., Muse cells were injected from the dura mater to the brain parenchyma using a Hamilton syringe. It was found that Muse cells may replace lost neurons by integrating into the peri-infarct cortex and spontaneously differentiating into neuron-labeled positive cells (Yamauchi et al., 2015). Similarly, the study in a mouse intracerebral hemorrhage (ICH) model was used in this way, and show that Muse cells can remain in the ICH brain, differentiate into neural lineage cells and restore function without inducing them to enter neuronal cells through gene introduction and cytokine therapy prior to transplantation (Shimamura et al., 2017). Additionally, Yabuki and Wakao (2018) show that Muse cells were injected into the pulmonary artery in the model of lung ischemia reperfusion, and more remained in the injured lung than MSCs, which improved the lung function and histological injury associated with IR injury in the rat model at the acute stage.

Therefore, no matter Muse cells are injected locally, injected into pulmonary artery or injected intravenously, as long as they migrate to the damaged site effectively, they will spontaneously differentiate into cells compatible with targeted tissues and secrete factors regulating microenvironment to promote tissue repair. It is known that in animal models of kidney, muscle, brain and liver injury, Muse cells will migrate to damaged tissues after local or systemic administration of drugs and spontaneously differentiate into histocompatibility cells to achieve the recovery of organ functions. Whether Muse cells were injected locally, into pulmonary arteries, or intravenously, there was no mention that Muse cells could be blocked from reaching the injury site due to pulmonary capillaries. It is known that in animal models of kidney, muscle (Kinoshita et al., 2015; Furuta et al., 2022), brain (Uchida H. et al., 2017; Shimamura et al., 2017) and liver injury (Nishizuka, 2018), Muse cells will migrate to damaged tissues after local or systemic administration of drugs and spontaneously differentiate into histocompatibility cells to achieve the recovery of organ functions. Thus, as long as they finally migrate to the damaged site effectively, they will spontaneously differentiate into cells compatible with the targeted tissue and secrete factors that regulate the microenvironment to promote tissue repair.

Microenvironmental cues at the injured site may play an important role in the fate decision of Muse cells. The microenvironment includes a variety of cytokines, growth factors, chemokines and extracellular matrix components. Then, in interaction with local factors, the lineage of Muse cells will be turned on or inhibited (Alessio et al., 2017; Alessio et al., 2018). Perhaps the reason Muse cells end up diverging in a particular direction can be explained by activating cellular and molecular signaling pathways that interact to form a complex regulatory network.

## 5 The regenerative potential of Muse cells has been confirmed

To date, Muse cells have been used in neurological diseases (Uchida H. et al., 2017; Shimamura et al., 2017; Hori et al., 2020; Ozuru et al., 2020; Chen et al., 2021; Kajitani et al., 2021; Suzuki et al., 2021; Yin et al., 2023), cardiac systemic diseases (Amin et al., 2018; Minatoguchi et al., 2018; Tanaka et al., 2018; Yamada et al., 2018; Yamada et al., 2022; Yamada et al., 2022; Castillo et al., 2023; Wu et al., 2023), renal diseases (Batchelder et al., 2009; Uchida N. et al., 2017; Uchida et al., 2018), dermatological diseases (Yamauchi et al., 2017; Yamauchi, 2018; Guo et al., 2020; Fei et al., 2021; Fujita et al., 2021; Fujita et al., 2021), liver diseases (Iseki et al., 2017; Nishizuka, 2018; Hessheimer et al., 2021b; Shono et al., 2021; Kikuchi et al., 2022), and other diseases to demonstrate their desirable effects in treating and repairing damaged tissues. In these disease models, Muse cells can migrate to the lesion site and spontaneously differentiate into histocompatible cells such as neurons (ectoderm) (Zheng et al., 2018; Hori et al., 2020; Yamashita et al., 2021), cardiomyocytes (mesoderm) (Noda et al., 2020; Yamada et al., 2022), glomerulocytes (mesoderm) (Batchelder et al., 2009; Uchida N. et al., 2017), vascular endothelial cells (mesoderm) (Kinoshita et al., 2015; Yamauchi, 2018) and hepatocytes (entoderm) (Iseki et al., 2017; Hessheimer et al., 2021b; Shono et al., 2021). Based on the good experimental results obtained before clinical practice, numerous clinical studies have evaluated Muse cells in treating conditions like stroke, myocardial infarction, spinal cord injury, neonatal cerebral palsy, dystrophic epidermolysis bullosa, and ALS (Alanazi et al., 2023). At present, CL 2020, a clinical-grade Muse cell product from Life Science Institute, Inc. featuring 1.5 × 10^7^ cells per 15 mL of frozen solution (Location: Tokyo, Japan). Due to the type of disease, the patient’s age, and constitution, clinical trial dosages vary and the appropriate amount is still being explored during treatment. We have summarized the available preclinical studies in Table 2.

**TABLE 2:** Preclinical studies using Muse cells for various diseases.

Category	Model of disease indications	Tissue source of Muse cells	Mechanisms for repairing damage	Administration method	References
Nervous system	Lacunar stroke	Bone marrow and CL 2020^a^	Differentiation	Intravenous administration and local injection	Uchida et al. (2017), Abe et al. (2020)
	Ischemic stroke	Bone marrow and dermal fibroblasts	Replenishment of neurons and oligodendrocytes, reconstruction of neuronal circuit	Intravenous administration and Local injection	Yamauchi et al. (2015), Uchida et al. (2016)
	Intracerebral Hemorrhage (ICH)	Bone marrow	Differentiation	Local injection	Shimamura et al. (2017)
	Amyotrophic lateral sclerosis (ALS)	Bone marrow	Differentiation	Intravenous administration	Yamashita et al. (2020)
	Neonatal hypoxic-ischaemic encephalopathy (HIE)	CL 2020^a^	Differentiation	Intravenous administration	Matsuyama et al. (2022)
	Spinal cord injury (SCI)	Bone marrow	Differentiation	Local injection	Chen et al. (2021)
	*E. coli*-associated encephalopathy	Bone marrow	Differentiation	Intravenous administration	Ozuru et al. (2020)
	Thoracic spinal cord contusion injury	Bone marrow and CL 2020^a^	Differentiation	Intravenous administration	Kajitani et al. (2021)
	Perinatal hypoxic-ischemic encephalopathy	Bone marrow	Regulation of glutamate metabolism and Reduction of microglial activation	Intravenous administration	Suzuki et al. (2021)
Cardiovascular system	Acute myocardial infarction (AMI)	Bone marrow and CL 2020^a^	Differentiation	Intravenous administration	Yamada et al. (2018), Yamada et al. (2022), Yamada et al. (2022)
Dermatosis	Epidermolysis bullosa (EB)	Bone marrow and CL 2020^a^	Differentiated into keratinocytes and functionally restored basement membrane zone (BMZ) proteins at the injury site	Intravenous administration	Fujita et al. (2021), Fujita et al. (2021)
	Diabetic skin ulcers	Adipose	Differentiation	Intravenous administration	Kinoshita et al. (2015)
	Atopic dermatitis	Bone marrow	Promote the migration and proliferation of keratinocytes	Subcutaneous injection	Fei et al. (2021)
	Corneal scarring wound	Abdominal lipoaspirate tissue	Increased corneal re-epithelialization and nerve regrowth, and reduced the severity of corneal inflammation and neovascularization	Placed with scaffold	Guo et al. (2020)
Kidney disease	Adriamycin Nephropathy	Bone marrow	Differentiation	Intravenous administration	Uchida et al. (2017)
Liver disease	Liver fibrosis	Bone marrow	Differentiation	Intravenous administration	Iseki et al. (2017)
	Post-hepatectomy liver failure	Bone marrow	Differentiation	Intravenous administration	Iseki et al. (2021)
Lung disease	Acute lung ischemia-reperfusion Injury	Bone marrow	secreted several substances involved in wound healing	Injected into pulmonary artery	Yabuki and Wakao (2018)
Another disease	Aortic aneurysms	Bone marrow	Differentiation	Intravenous administration	Hosoyama et al. (2018)
	Acute pancreatitis	Bone marrow	attenuating edema, inflammation and apoptosis	Intravenous administration	Fukase et al. (2022)
	Cartilage lesions	Bone marrow	Differentiation	Intravenous administration	Mohamed-Ahmed et al. (2018)
	Cartilage defects	Synovial tissue and Bone marrow	Differentiation	Intravenous administration	Alessio et al. (2017), Toyoda et al. (2019)
	Intestinal inflammatory diseases	Bone marrow	Anti-inflammatory and immune regulatory functionality	Intravenous administration	Sun et al. (2020)
	Hunner-type interstitial cystitis (HIC)	Bone marrow	Paracrine effect	Injected into the anterior and posterior bladder wall	Furuta et al. (2022)
	Diabetes mellitus	Adipose	Paracrine effect	Intravenous administration	Fouad et al. (2018), Perone et al. (2018)

^a^ CL2020: The clinical-grade Muse cell–based product CL2020 was produced from human MSCs by exposing the cells to the combination of stresses and were confirmed to be positive for both pluripotency marker SSEA-3; Semi-clinical grade human Muse cell preparation was provided by Clio, Inc. (merged into Life Science Institute, Inc. Tokyo).

### 5.1 The role of Muse cells in nervous system diseases

Currently, it is crucial to develop treatments that promote neurological recovery and rebuild damaged neural circuits. Neurological related diseases face a variety of obstacles and challenges. Lack of neuronal regenerative capacity leads to disability and death in many neurological disorders including stroke, and more typically neuroinflammation impedes central nervous system (CNS) repair, i.e., a massive loss and ineffective replenishment of neuronal cells, resulting in difficulty in regeneration of damaged neural tissue (Fawcett, 2020). Microglia are important immune cells in the CNS, which can be divided into 2 cell types, M1 and M2, and are sensitive to changes in the external environment, affecting the status of surrounding astrocytes and neuronal cells and regulating the immune response in the vicinity (Ransohoff, 2016; Mosser et al., 2017; Vidal-Itriago et al., 2022). Recent studies have found that Muse cells reduce neuroinflammatory responses *in vitro* by regulating the ratio of M1-type to M2-type microglia, possibly by inhibiting the small TLR4/MyD88/NF-κB and p38 MAPK signalling pathways in microglia to exert anti-neuroinflammatory effects, providing new ideas for further application of Muse cells in the treatment of CNS diseases and injuries (Yin et al., 2023).

Among the available studies, Human BM-Muse cells cultured in serum-free/allogeneic medium were transplanted into an immunodeficient mouse model of lacunar cerebral infarction for 2 weeks, and it was found that after 8 weeks, approximately 28% of the initially transplanted Muse cells remained in the host brain and spontaneously differentiated into cells expressing NeuN (∼62%), MAP2 (∼30%), and GSTpi (∼12%), and the final results showed that the model mice recovered their neurological function well, and the transplanted Muse cells differentiated into neurons and oligodendrocytes and participated in the reconstruction of cone fascicles, and have a favorable safety profile (Uchida H. et al., 2017). Moreover, experiments with middle cerebral artery occlusion in immunodeficient mice demonstrated that Muse cells incorporated into the peri-infarct cortex were able to spontaneously differentiate into cells positive for the neuronal markers Tuj-1 (45.3% ± 13.9%) and NeuN (20.5% ± 8.7%), replenishing lost neurons and thus restoring motor function (Yamauchi et al., 2015). In a mouse model of ICH, Muse cells can integrate into the region of cerebral vascular injury and differentiate into Neu N- and MAP-2-positive neurons, improving survival and motor function (Shimamura et al., 2017). In addition, amyotrophic lateral sclerosis (ALS) (Yamashita et al., 2020), and E. coli-associated encephalopathy (Ozuru et al., 2020), Muse cells have therapeutically mitigated the lethality of the disease and facilitated tissue repair through spontaneous differentiation into neurons and neuroglia after homing into the damaged central nervous system.

It’s worth noting that in phase I, the single-center, open-label, dose-escalation clinical trial employing CL2020 evaluates the safety and tolerability of CL2020 cells in treating hypoxic-ischaemic encephalopathy (HIE) in newborns undergoing hypothermia therapy (ClinicalTrials.gov Identifier: NCT04261335, jRCT2043190112). Effective treatments for stroke after the acute phase remain elusive, but the efficacy of CL2020 as a treatment for subacute ischemic stroke was shown in a placebo-controlled, randomized study—details in the registry: JAPIC Clinical Trials Information site (JapicCTI-184103) (Niizuma et al., 2023). The use of Muse cells promises to be an effective means of treating CNS disorders. Seed cells that both promote nerve regeneration and improve the CNS microenvironment.

### 5.2 The role of Muse cells in cardiovascular system diseases

Using Semi-clinical grade human Muse cell product in the Swine model of acute myocardial infarction, Muse cells homed to the infarct margins and differentiated into cardiomyocytes (troponin I-positive) and microvessels (CD31-positive), which were able to reduce the size of the infarcts and improve ventricular function and remodeling (Yamada et al., 2022). In an acute myocardial infarction model, Muse cells homed to post-infarct tissue and spontaneously differentiated within 2 weeks into cells positive for cardiomyocyte markers such as troponin-I, α-actinin and connexin 43 proteins, exhibiting calcium inward and outward currents synchronized with electrocardiogram-recorded cardiac activity, and the expression of MLC 2a (Myosin Light Chain 2a) and MLC 2v (Myosin Light Chain 2v), which also demonstrated the ability of Muse cells to differentiate into atrial and ventricular cardiomyocytes (Amin et al., 2018). During a myocardial infarction clinical study (Japic CTI-183834 and Japic CTI-195067), administering CL2020 intravenously (Life Science Institute, Inc., Tokyo, Japan) proved to be safe, enhancing left ventricular ejection fraction and wall motion score index (Noda et al., 2020).

In conclusion, Muse cells are capable of spontaneous differentiation into cardiac and vascular lineages, and additionally have extraordinary potential for the treatment of cardiovascular disease through regeneration of cardiomyocytes and blood vessels, as well as paracrine effects that more dramatically reduce the size of myocardial infarcts and improve cardiac function (Yamada et al., 2022).

### 5.3 The role of Muse cells in skin regeneration

Transplantation of Muse cells promotes reconstruction of damaged skin tissue by replenishing new dermal and epidermal cells. In a report of skin ulcers in type 1 diabetic immunodeficient mice, Muse cell-treated ulcers showed faster healing with thicker epidermis (Kinoshita et al., 2015). In addition, after induction of Muse-AT cells into fibroblasts, keratinocytes and melanocytes, skin sheets were reconstructed by these differentiated cells and collagen gel layers, and the reconstructed hyperpigmented skin formed an epidermal-like structure (Tsuchiyama et al., 2013). A non-randomized, single-arm, non-controlled clinical trial (Japic CTI-184563) was chosen to examine dystrophic epidermolysis bullosa in patients experiencing recurrent and refractory ulcers for over 4 weeks. Fascinatingly, the CL2020 infusion enhanced the area affected by skin erosion and decreased the size of ulcers (Fujita et al., 2021; Fujita et al., 2021). Thus, transplantation of Muse cells may be an effective treatment for skin-related diseases.

### 5.4 The role of Muse cells in liver diseases

Muse cells are integrated as hepatic progenitor cells in the early stage, and then spontaneously differentiate into major liver components such as hepatocytes, bile duct cells, sinusoidal endothelial cells and Kupffer cells in the physical partial hepatectomy model (Iseki et al., 2021). In a mouse model of liver injury, human Muse cells expressing CK19, DLK, OV6 and alpha-fetoprotein (markers of hepatic progenitor cells) 2 days after intravesical injection and expressing the mature hepatocyte markers HepPar1, albumin and antitrypsin within 2 weeks had a very high homing rate in damaged livers and stayed in host tissues for 8 weeks, integrating briefly by intravenous injection into the damaged liver, spontaneously differentiated into hepatocytes *in vivo*, and finally significantly improved liver function in model mice by attenuating fibrosis (Iseki et al., 2017). Postoperative liver failure (PHLF) is a potentially fatal complication. The safety and efficacy of transapical infusion of allogeneic Muse cells in a porcine model of PHLF was assessed. Specific homing of Muse cells to the liver resulted in improved control of hyperbilirubinaemia, the international normalized ratio of prothrombinogen (*p* = 0.05), and suppression of focal necrosis. The integrated Muse cells spontaneously differentiated into hepatocyte marker-positive cells. Muse cell transplantation may provide a reparative role and functional recovery in a hepatic resection model and thus may contribute to the treatment of PHLF (Nishizuka, 2018; Iseki et al., 2021).

These studies suggest that Muse cells are a viable stem cell type for the treatment of liver disease.

### 5.5 The role of Muse cells in another disease (including lung injuries, kidney disease, osteochondral defects)

Muse cells have also been highlighted in other diseases as follows. Human Muse cell administration improved lung function and histological damage associated with acute phase ischemia-reperfusion injury in a rat model. Muse cells were more abundant in lung tissue from ischemia-reperfusion injury compared to MSCs. Human Muse cells secreted beneficial substances (KGF, HGF, Ang-1, and PGE 2) *in vitro*, and it is possible that these protective factors together exert tissue repair, apoptosis prevention and alveolar fluid clearance (Yabuki and Wakao, 2018; Yabuki and Watanabe, 2018; Chen et al., 2022). Muse cells also have therapeutic potential for osteochondral repair (Alessio et al., 2017). Osteochondral defects were produced in the patellar groove of immunodeficient rats and intra-articular injected with Muse cells. At 12 weeks, the Muse defects were completely filled with smooth homogenous tissue, which made it difficult to clearly identify the defect edges. Although the repaired tissue in the Muse group was negative for type II collagen, indicating unsatisfactory cartilage repair, histological scoring showed better subchondral bone repair at the site of the cartilage defect. Extensive studies on the cartilage-forming potential of Muse cells are needed. Muse cells have great potential for the treatment of inflammatory bowel disease and other inflammatory diseases of the gut (Sun et al., 2020).

In conclusion, it is worth keeping an eye on the powerful regenerative capacity of Muse cells, and I believe there are many more promising therapeutic effects in the pipeline!

## 6 Future prospects and challenges

Muse cells have features that compensate for the shortcomings of current stem cells such as iPSCs and ESCs. These cells are naturally occurring reparative stem cells in the body that do not cause tumors, have the ability to differentiate spontaneously, and can be targeted to damaged tissues through the S1P-S1PR2 axis. Furthermore, Muse cells do not require gene introduction or cytokine induction to present pluripotency or induce differentiation into the cell type of interest prior to clinical use before treatment, and it is an expedient for patients to provide viable regenerative therapy through intravenous infusion. Even though Muse cells have practical advantages for regenerative medicine, there are still unresolved difficulties and some unknown challenges. Although Muse cells naturally exist as endogenous cells rather than immortalized or monoclonal expanded tumorigenic cells, the homogeneity of Muse cells remains unknown. Additionally, Muse cells account for only a small population of various sources and take time to expand to enough cells for clinical administration. Take stroke as an example, the time window is narrow (Lin et al., 2016; Campbell et al., 2019), and patients cannot use freshly prepared Muse cells.

In particular, compared with MSCs, the culture cost of Muse cells is more expensive and the culture procedure is more complex. Moreover, complex steps will introduce more variable parameters, resulting in inconsistent product quality of different batches of cells, and ultimately lead to different treatment outcomes after clinical transplantation. At present, Muse cells clinical trials are limited for diseases. In view of the above-mentioned facts, Muse cells are in urgent need of gold standardization, to establish a strict GMP compliance process, to provide patients with high quality, high consistency Muse cells in urgent need of gold standardization. Furthermore, the complex mechanisms and pathways by which Muse cells differentiate into histocompatibility cells are still far from being fully understood. Therefore, it is necessary to further investigate which signaling pathways or transcription factors control the differentiation of Muse cells into specific directions. For example, by introducing the relevant genes into Muse cells or modifying them with nanomaterials, it may be possible to increase the rate of differentiation into the intended cell line after transplantation to the damaged site. Tissue-engineered MSCs are a paradigm of MSCs associated with biomaterials, either a scaffold or a hydrogel. Still, it's worth noting that the existing research has found that MSCs impregnated scaffolds/patches and MSC secreted exosomes, such patches can be seen as a future approach to limit the ROS levels (Raghav et al., 2022). It's possible that Muse cells are capable of optimally mending damaged tissue using this method.

Its impressive regenerative properties may provide a simple and feasible strategy to treat a variety of diseases. The Muse cell is being used as a delivery system that may play a role in improving the delivery of drugs and lysosomal viruses to recalcitrant tumors, and may also be considered for engineering into molecules with angiogenic, neurotrophic and anti-inflammatory properties to accelerate the repair of damaged or diseased tissues. Therefore, the unique properties of Muse cells and their great potential in repairing damage need further research and development.

## Figures and Tables

**Table:** 

**Mus**e	Multilineage-differentiating stress-enduring	
**S1P**	sphingosine-monophosphate
**S1PR2**	sphingosine-monophosphate receptor 2
**MSCs**	mesenchymal stem/stromal cells
**BM**	bone marrow
**SSEA-3**	Stage-specific embryonic antigen 3
**FACS**	fluorescence-activated cell sorting
**MACS**	magnetically-affinitive cell sorting
**PB**	peripheral blood
**AT**	adipose tissue
**UC**	umbilical cord
**BMMSCs**	bone marrow-derived mesenchymal stromal cells
**UC -MSCs**	umbilical cord-derived mesenchymal stromal cells
**MenSC**	menstrual blood-derived endometrial stem cells
**FACS**	fluorescence-activated cell sorting
**OCT3/4**	octamer binding transcription factor 3/4
**SOX2**	sex-determining region Y box 2
**REX1**	reduced expression 1
**DDR**	DNA damage response
**LIF**	leukemia inhibitory factor
**BMP4**	bone morphogenetic protein 4
**AMI**	acute myocardial infarction
**HLA**	human leukocyte antigen
**MHC**	major histocompatibility complex
**HLA-G**	Human leukocyte antigen G
**IDO**	Indoleamine 2,3- dioxygenase
**Treg**	regulatory T cells
**AD-MSCs**	adipose-derived mesenchymal stromal cells
**IR**	ischemia-reperfusion
**TNF-α**	tumor necrosis factor-α
**S1P**	sphingosine-1-phosphate
**S1PR2**	specific G-protein-coupled receptors receptor 2
**AMI**	acute myocardial infarction
**ICH**	intracerebral hemorrhage
**CNS**	central nervous system
**ALS**	amyotrophic lateral sclerosis
**PHLF**	Postoperative liver failure
**HIE**	hypoxic-ischemic encephalopathy
**SCI**	spinal cord injury
**EB**	Epidermolysis bullosa
**BMZ**	basement membrane zone
**HIC**	Hunner-type interstitial cystitis
